# Whole genome profiling of spontaneous and chemically induced mutations in *Toxoplasma gondii*

**DOI:** 10.1186/1471-2164-15-354

**Published:** 2014-05-10

**Authors:** Andrew Farrell, Bradley I Coleman, Brian Benenati, Kevin M Brown, Ira J Blader, Gabor T Marth, Marc-Jan Gubbels

**Affiliations:** Department of Biology, Boston College, Higgins Hall 355, 140 Commonwealth Avenue, Chestnut Hill, MA 02467 USA; Department of Microbiology and Immunology, University of Oklahoma Health Sciences Center, 940 Stanton L. Young Blvd., BMSB 1053, Oklahoma City, OK 73104 USA; Department of Molecular Microbiology, Washington University School of Medicine, MPRB 9230, 4940 Parkview Place, St. Louis, MO 63110 USA; Department of Microbiology and Immunology, State University of New York, 138 Farber Hall, 3435 Main Street, Buffalo, NY 14214 USA

**Keywords:** Whole genome sequencing, Chemical mutagenesis, *In vitro* adaptation, SNV calling, Apicomplexa

## Abstract

**Background:**

Next generation sequencing is helping to overcome limitations in organisms less accessible to classical or reverse genetic methods by facilitating whole genome mutational analysis studies. One traditionally intractable group, the Apicomplexa, contains several important pathogenic protozoan parasites, including the *Plasmodium* species that cause malaria.

Here we apply whole genome analysis methods to the relatively accessible model apicomplexan, *Toxoplasma gondii*, to optimize forward genetic methods for chemical mutagenesis using N-ethyl-N-nitrosourea (ENU) and ethylmethane sulfonate (EMS) at varying dosages.

**Results:**

By comparing three different lab-strains we show that spontaneously generated mutations reflect genome composition, without nucleotide bias. However, the single nucleotide variations (SNVs) are not distributed randomly over the genome; most of these mutations reside either in non-coding sequence or are silent with respect to protein coding. This is in contrast to the random genomic distribution of mutations induced by chemical mutagenesis. Additionally, we report a genome wide transition vs transversion ratio (ti/tv) of 0.91 for spontaneous mutations in *Toxoplasma*, with a slightly higher rate of 1.20 and 1.06 for variants induced by ENU and EMS respectively. We also show that in the *Toxoplasma* system, surprisingly, both ENU and EMS have a proclivity for inducing mutations at A/T base pairs (78.6% and 69.6%, respectively).

**Conclusions:**

The number of SNVs between related laboratory strains is relatively low and managed by purifying selection away from changes to amino acid sequence. From an experimental mutagenesis point of view, both ENU (24.7%) and EMS (29.1%) are more likely to generate variation within exons than would naturally accumulate over time in culture (19.1%), demonstrating the utility of these approaches for yielding proportionally greater changes to the amino acid sequence. These results will not only direct the methods of future chemical mutagenesis in *Toxoplasma*, but also aid in designing forward genetic approaches in less accessible pathogenic protozoa as well.

**Electronic supplementary material:**

The online version of this article (doi:10.1186/1471-2164-15-354) contains supplementary material, which is available to authorized users.

## Background

The Apicomplexa comprise important human pathogens such as the malaria-causing *Plasmodium* spp. [1] and *Toxoplasma gondii*, which can cause life-threatening opportunistic disease and birth defects [2]. Due to the complex life cycle of most Apicomplexa, the experimental accessibility of these parasites has been limited. However, *Toxoplasma* is a comparatively accessible model for other apicomplexan parasites [3]. The development of *Toxoplasma* as a forward genetic system was pioneered in the 1970s by Elmer Pfefferkorn, who took advantage of the ability to culture the asexual tachyzoite life cycle stage indefinitely *in vitro* and the parasite’s short generation time of ~7 hrs. Using mutagenized parasites Pfefferkorn started to dissect the nucleotide salvage and synthesis pathways ( [4, 5] reviewed in [6]). In the years since Pfefferkorn’s original work, chemical mutagenesis and forward genetic analyses have been successfully applied to various unique aspects of *Toxoplasma* biology, including invasion and egress from the host cell and internal budding, the parasite’s distinct mode of cell division [6–14].

Full exploitation of the power of *Toxoplasma* forward genetics will require a better understanding of the mutagenic profiles associated with specific mutagenesis protocols. Pfefferkorn initially used N-ethyl-N-nitrosourea (ENU) on actively growing intracellular parasites. Efficient mutagenesis was also obtained through nitrosoguanidine treatment of extracellular parasites and ethylmethane sulfonate (EMS) treatment of intracellular parasites [15]. ENU and EMS are both widely used chemical mutagens: EMS is favored in genetic studies in plants [16], fruit flies [17] and *C. elegans*[18], whereas ENU is preferentially used in mice [19] and zebrafish [20]. The choice of mutagen is dependent upon the organism’s DNA composition, DNA repair pathway efficiencies, and the chemical’s mutagenic signature. As a rule of thumb, EMS preferentially results in G/C base pair mutations whereas ENU has a bias toward A/T base pairs [21–23]. We previously confirmed the A/T proclivity of ENU in a single *Toxoplasma* mutant [7], but the EMS mutagenic signature is currently unknown.

Whole genome sequencing (WGS) has been used in model organisms such as *Caenorhabditis elegans*[18] and *Saccharomyces cerevisiae*[24, 25] to identify the numbers of mutations per genome, the ratio of silent vs. non-silent mutations, the chance of generating mono-allelic traits and other genome-wide analyses. By cataloging the mutations in a single genome associated with a specific phenotype, these approaches also allow mapping of phenotypic traits to genomic loci. This has been best explored and defined in *C. elegans*[18, 24, 26–29], but there are examples from zebrafish [30] and *S. cerevisiae*[25, 31] as well. WGS and analysis of the genome-wide distribution of mutations are new tools whose power has been recognized in genetic model organisms [32]. In developing genetic systems for non-model organisms, however, this power has not been fully exploited.

In *Toxoplasma,* the combination of forward genetics and WGS has extended Pfefferkorn’s pioneering chemical mutagenesis studies to map drug resistance genes [33, 34] and biological phenotypes in invasion and egress [7, 13]. To further the development of the *Toxoplasma* genetic system, we previously initiated an optimization of forward mutagenic protocols [35]. Here we further exploit the power of WGS to expand on these efforts by defining the mutagenic profiles of ENU and EMS at varying dosages. Through the analysis of 1208 single nucleotide variations (SNVs) spontaneously generated in *Toxoplasma* under lab conditions, we also show that these mutations reflect genome nucleotide composition, without any bias. Furthermore, we show that both ENU (369 SNVs) and EMS (158 SNVs) have a proclivity for inducing mutations at A/T base pairs while also generating greater proportions of protein code changing SNVs than those generated spontaneously during *in vitro* culture. Finally, we show there are no apparent hot- or cold-spots within the genome for variations generated via either *in vitro* culture or chemical mutagenesis. We use these insights to design an optimized chemical mutagenesis protocol for forward genetic experiments in *Toxoplasma* and potentially other Apicomplexa.

## Methods

### Parasites

**Figure 1:**
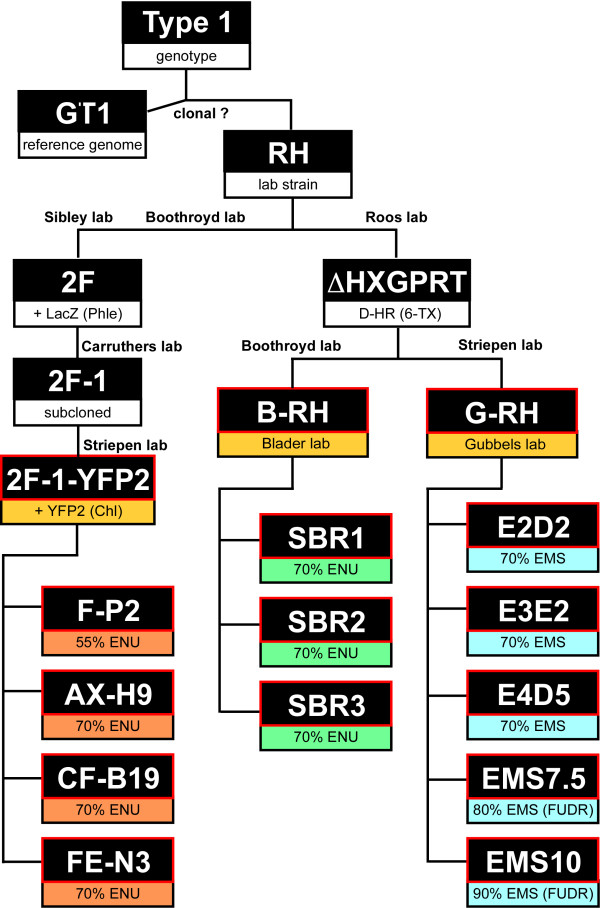
**Lineage overview of the parasite strains used in this study.** All are of the Type I genotype, with GT1 the sequenced reference genome available on ToxoDB.org [48]. The GT1 and RH strains are most likely descendants from the same clone [49]. All sequenced strains and mutants are outlined by red boxes; all are derived from the RH strain. Drug selections to generate transgenic lines are given between brackets in the lower box of each line. Mutagen dosage is expressed as the percentages of parasites being killed by mutagen treatment (measured by plaque assays). Phle: phleomycin; D-HR: double homologous recombination; 6-TX: 6-thioxanthine; Chl: chloramphenicol; FUDR: 5-fluoro-2'-deoxyuridine; HXGPRT: hypoxanthine-xanthine-guanine-phosphoribosyl transferase.

Parasites were maintained by *in vitro* passage in human foreskin fibroblasts (HFF cells) [36]. An overview of the genealogy of these strains is given in Figure 1. All the strains are derived from the Type I RH *Toxoplasma* strain isolated from a 1939 case of toxoplasmic encephalitis [37] and subsequently cloned and adapted for *in vitro* culture in the 1970s [5]. The RH-HXGPRT knock-out strain (RH-ΔHXGPRT) was generated by homologous recombination and 6-thioxanthine selection in the 1990s [38]. The RH-ΔHXGPRT strain made its way from the Roos lab to the Boothroyd and Striepen labs, and from there to the Blader and Gubbels labs, respectively. For the purpose of this paper these sibling strains are referred to as the ‘B-RH’ and ‘G-RH’ strains. The transgenic 2F line stably expresses LacZ (β-galactosidase), which was selected for stable, random genomic integration by phleomycin through the BLE selectable marker [39]. The 2F line was recloned around 2000 by Vern Carruthers to make 2F-1 (also referred to as 2F1 [40]). Subsequently, chloramphenicol selection for the CAT selectable marker was applied to stably integrate a tandem YFP expressing plasmid resulting in 2F-1-YFP2 [41]. The number of passages (each representing ~8 generations) along the journeys of these strains is unknown, but is likely in the range of 100-1000s.

### Mutagenesis and phenotype screening

Chemical mutagenesis was performed essentially as described in [35]. Briefly, a T25 flask of confluent HFFs was inoculated with 1 ml of freshly lysed *T. gondii,* which were allowed to invade and replicate in Ed1 media for 18–25 hrs at 37°C. Ed1 was replaced with 10 ml of 0.1X Ed1 (diluted in DMEM) and the flasks returned to 37°C for 10 minutes. Intracellular parasites were treated with the mutagen of choice and the appropriate vehicle controls for 4 hrs at 37°C. After exposure to mutagen, parasites were recovered by washing the monolayer 3X with cold PBS, scraping and lysing the host cells via passage through a 26.5G needle and filtration through a 3.0 μm polycarbonate filter. Parasites were recovered by centrifugation at 1000×g for 10 min and returned to a new confluent T25 flask. The mutagenized population was allowed to lyse the monolayer naturally, typically in 5–7 days. Percent killing was determined by serial dilution of 10,000 mutagenized and vehicle treated parasites into 6 well plates confluent with HFF cells. After 1 week of undisturbed growth, wells were stained for the presence of plaques. Percentages were calculated as a ratio of plaque number in treated parasites vs. the vehicle control. Following phenotypic selection of mutants, individual parasite clones were isolated through serial dilution in 384 well plates of confluent HFFs. After 7 days, wells containing only single plaques, as seen through the microscope, were considered monoclonal populations.

ENU mutants were generated in different genetic backgrounds at concentrations of ENU in the range of 3–7 mM (Figure 1; because ENU is unstable, stocks should be titrated relative to percent killing as described above [35]). Mutants F-P2, AX-H9, CF-B19, and FE-N3 were generated by Gubbels and screened for a temperature sensitive growth defect (permissive and restrictive temperatures were 35°C and 40°C, respectively) [8]; mutants SBR1-3 were generated by the Blader lab and screened for resistance against the kinase inhibitor SB505124 [33]. Mutant F-P2 was generated using a 55% killing dose; for all others a dose inducing 70% killing was used. All mutants were generated by independent mutagenesis experiments and therefore represent unique SNV pools. For this study we resequenced mutant F-P2 with longer reads. We differentiate the “old” data from the “new” data as o-F-P2 [7] and n-F-P2. The two DNA isolations were performed two passages apart, which corresponds to approximately 16 generations.

EMS mutants were obtained at mutagen dosages ranging from 3–10 mM in the G-RH genetic background. Again, as above, the mutagen was titrated via percent killing. Mutants E2D2, E3E2, and E4D5 were generated at 70% killing. E2D2 and E4D5 were screened for resistance against DTT induced egress [42] whereas E3E2 was screened for resistance against the invasion enhancing compound 2 [42, 43]. Mutants EMS7.5 and EMS10 were generated using 7.5 and 10 mM EMS, inducing 80% and 90% killing, respectively and screened for resistance against 20 μM FUDR [15]. All EMS mutants were also generated by independent mutagenesis experiments.

### Whole genome sequencing

Parasites were collected 24 hrs after complete lysis of the HFF cell monolayer without further scraping, passed three times through a 26.5G needle, and filtered through a 3 μm filter membrane. Harvesting parasites 24 hrs post host cell lysis, while taking care not to disturb the HFF monolayer, largely eliminates the 50% host DNA background we previously observed [7]. Genomic DNA was prepared with the Qiamp DNA mini kit (Qiagen; Valencia, CA) using the manufacturer’s protocol for cultured cells. Illumina sequencing of strains 2F-1-YFP2 and o-F-P2 was performed at the Broad Institute, Cambridge, MA as described in [7] and all others at the Arthritis & Clinical Immunology Department of the Oklahoma Medical Research Foundation, Oklahoma City, OK as described in [33].

### SNV calling

Methods used here are essentially the same as have been previously described [7]. FASTQ sequence traces were aligned to a FASTA reference containing both the *Toxoplasma gondii* GT1 genomic reference v7.0 and the human genome reference build 37. Alignments were performed with MOSAIK using -mmp .15, -mhp 100 -act 35 -hs 15. Variable descriptions and procedures are described in the documentation V1.0 (available at http://bioinformatics.bc.edu/marthlab/wiki/index.php/Software) [44]. Variations were called with FreeBayes using standard parameters as described in the documentation, software version 0.9.9 [45, 46]. The resulting SNV calls for the respective samples were compared to the SNV calls for its respective parent sample to identify both variations that were shared between the two samples and those that were unique to the mutant. Calls were filtered to remove SNVs with coverage less than 5X in either sample, a P value less than 0.8, and less than 75% allele balance in either samples at that position.

To compare the three parent samples, 2F-1-YFP2, B-RH, and G-RH, each parent was compared to each of the other two parents separately as described above, creating a total of 3 datasets: n-F-P2 vs. B-RH, n-F-P2 vs. G-RH, and B-RH vs. G-RH (note that n-F-P2 sequence data was used instead of 2F-1-YFP2 since its coverage is greater and the error rate and human contamination are much lower; F-P2 specific mutations [7] were manually removed after analysis). In each of the samples shared and unique SNVs from each comparison were pooled together to create a complete list of variants for each sample. To create a high confidence unified set for three samples, the rejected positions for all three comparisons were pooled and SNV calls that intersected with any rejected position were removed from the variant lists.

### Other computational methods

Circose plots were generated using Circos as described in [47]. *Toxoplasma* gene densities were calculated using GFF data for GT1 v7.0 available from http://toxodb.org[48]. Percentages were calculated by counting the number of bases within regions annotated as “gene” in a 100 kb window.

### SNV validation

PCR primers were designed to amplify 250 bp up- and down-stream of a select number of called mutations (further details and primer sequences provided in Additional file 1: Table S1). Primers were designed using an automated algorithm that required primers to fit the following description: total product size of 500 ± 200 base pairs, primer GC content between 40% and 50%, the 3’ most base must be either a G or C, of the last five 3’-basepairs three must be either A or T, and the primer must be unique within the *Toxoplasma* genome. Primers are initially designed as 20 bases, if a primer could not be found that satisfied the above criteria at that length the calculations were run again with 19 bases, then 21 bases and so on. Furthermore, the sense primers were designed with a 5’ extension composed of the M13 universal primer. PCR products were amplified from parent and mutant genomic DNA and analyzed by agarose gel electrophoresis. PCR bands were extracted from gel and sequenced using the M13 universal primer.

## Results

### Sequencing results

**Figure 2:**
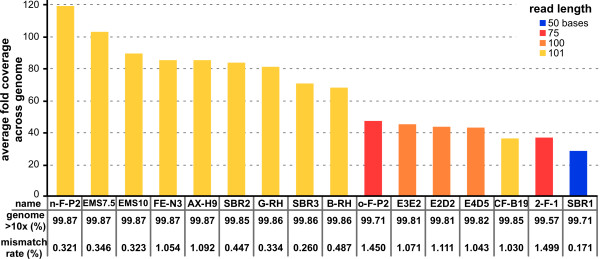
**Genomic coverage and quality of sequencing reads.** Average fold genomic coverage for the various strains is plotted as indicated. Bar color reflects the Illumina read length used as indicated in the legend. In the Table in the lower half the percentage of the genome covered at least 10-fold is shown as percentage of the complete GT1 reference genome (in all cases over 99.5%). Read quality is reported as the mismatch rate between reads and the reference genome. n-F-P2 [n = new] is a re-sequenced sample of the F-P2 mutant with longer read length [7], named o-F-P2 [o = old].

Illumina paired-end whole genome sequence reads were obtained for all strains outlined by red boxes in Figure 1. Collected reads were aligned to the *Toxoplasma* GT1 reference strain genome assembly [48]. The GT1 strain was isolated from goat skeletal muscle and is a Type I genotype strain closely related to the RH strain, for which no fully sequenced reference currently exists [49]. Sequences were obtained on different machines in different facilities, producing different read lengths and qualities (Figure 2). Regardless of differences in read length, genomic coverage was very high with more than 99.5% of the reference genome covered at a read depth of 10 or greater. Average genome coverage ranged from a depth of 29 for SBR1 to 119 for n-F-P2. From a practical perspective it is important to cover as much of the genome as possible when looking for a causative mutation. Our alignment covers >99.5% of the sequenced GT1 reference, but because gaps remain in the completed sequence, parts of the genome might be absent from our analyses. To assess how much of the genome may be missing from the reference we identified the proportions of our total reads that aligned to the 14 chromosomal contigs, to the 365 unplaced contigs or were unaligned. Less than 1% of the reads aligned to the 365 unaligned contigs, implying that these contigs do not account for more than 1% of the true genome. Between 2% and 10% of the sequence reads could neither be aligned to the GT1 reference, nor to the human (host cell) genome. A significant percentage of unaligned reads is common and expected in Illumina sequence; these reads likely represent simple sequencing errors, not significantly large missing parts of the RH genome. Previous studies report alignment percentages from as low as 70% up to 94% [24, 27, 50–52]. We therefore estimate that the 14 complete contigs in the *Toxoplasma* reference likely comprise as much as 99% of the true genome. Whether the remaining 1% represents a true chromosomal discrepancy between GT1 and RH is not further pursued here.

### SNV calling

We called SNVs in all samples using FREEBAYES. Our previous work has shown that numerous areas in the GT1 reference appear to be either duplicated or assembled incorrectly when compared to our RH strains. This produces incorrect read alignments and leads to a high rate of spurious variant calls. As we previously published, to filter out potentially spurious SNV calls in these regions, mutant samples were compared to the relevant parent line to remove areas that had poor alignments in either sample [7]. In that work, after filtering there were 997 polymorphisms shared between the o-F-P2 and 2F-1-YFP2 parent, and 33 candidate mutations unique to F-P2. Fifteen of the SNVs removed by filtering were randomly selected and all were experimentally confirmed as false positives. 31 of 33 mutations called as unique in mutant F-P2 (using the o-F-P2 data) were validated by PCR amplification and Sanger sequencing [7]. Of the two remaining false positive calls, one was eliminated by using the n-F-P2 sequence reads produced for this study, which are of lower error rate, deeper coverage and longer read length. For data generated in this study, we validated a select number of SNV calls in EMS mutants E2D2, E3E2, and E4D5 (Additional file 1: Table S1). The data for 30 variations identified no false positive calls. Hence, the false positive rate based on these sets of data is 1 out of 63 SNVs (33 + 30 total tested), which is 1.58%. Although the false positive rate is low, and may represent an overestimation given that no false positives were confirmed in the new dataset, it should be considered in the interpretation of the presented data.

To identify differences between the three parental strains each sample was compared against the two other parent samples. For each strain, the mutations (relative to the reference) that were found only in that parent were combined with those mutations shared amongst the parental strains to create a complete high quality set of SNVs for that sample. To ensure equal confidence in the data from all three samples, SNVs occurring at positions rejected in any of the samples were removed from all three mutants. This is a more conservative set of calls than the paired set, as any reduction in coverage in one sample will affect calls in all three, but ensures an unbiased comparison between the three samples. The parent sample 2F-1-YFP2 has by far the highest mismatch rate and second lowest coverage of all of samples. To improve the quality of the parent comparison, the well characterized sample n-F-P2 [7], of which 2F-1-YFP2 is the parental strain, was substituted and the 31 confirmed SNVs caused by ENU mutagenesis were removed. SNV calls and further analyses for all strains and mutants are available in Additional file 2: Table S2.

### Natural variations in non-mutagenized parent laboratory strains

**Figure 3:**
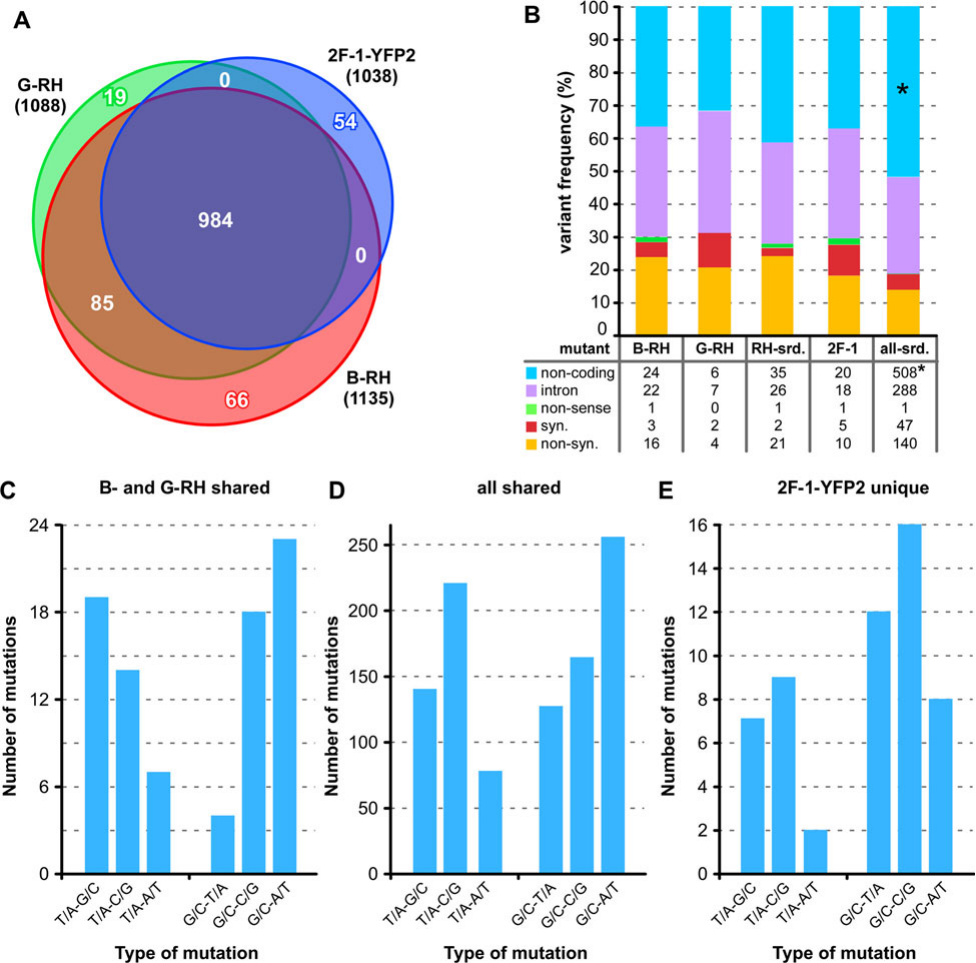
**Comparison of non-mutagenized parent strains: 2F-1-YFP2, Blader RH-ΔHGXPRT (B-RH) and Gubbels RH-ΔHGXPRT (G-RH). (A)** Venn diagram of shared and unique SNVs between the three strains and the GT1 reference genome. **(B)** The incidence of various mutations causing changes in amino acid coding. For 2F-1-YFP2, B-RH, and G-RH the unique SNVs vs GT1 are shown (these correspond to the 54, 66, and 19 SNVs in panel A, respectively). “RH-srd.” refers to all SNVs shared between B-RH and G-RH and “all-srd.” refers to the SNVs shared between all three lines (these correspond to the and 85 and 984 SNVs in panel **A**, respectively) Syn.: synonymous; non-syn: non-synonymous. * 50 non-coding SNVs map to the apicoplast genome. **(C-E)** The incidence of the various base pair changes (SNVs) in the mutants and groups as indicated. Note that we used the n-F-P2 reads instead of 2F-1-YFP2 reads in this analysis since the quality is much higher (Figure 2); ENU specific n-F-P2 mutations were removed from the comparative analysis.

Reads of the three RH-derived parent strains are analyzed here: 2F-1-YFP2, B-RH and G-RH (Figure 1). Compared to GT1, 984 mutations are shared among all three (50 of these are in the apicoplast genome). In this dataset, the rate of transitions (i.e. mutations of T/A to C/G and from G/C to A/T) to transversions (T/A to G/C, T/A to A/T, G/C to T/A or G/C to C/G) was 0.92 (Figure 3). The ti/tv rate is unique for each species and possibly reflects the nature of DNA repair activity in the species [53]. Because of the wobble base pairing of codon and anti-codon in the ribosome, transitions result in more radical amino acid changes than transversions [54]. These variations could be the result of differences between the primary RH and GT1 isolates, but are more likely to have accumulated during the extensive lab maintenance of the RH strain before these three parental lines diverged. In addition to the single nucleotide mutations shared among all three lines in our dataset, the sibling RH-ΔHXGPRT strains share an additional 85 SNVs not found in 2F-1-YFP2. Beyond this, B-RH contains 66 completely unique SNVs while G-RH has 19. 2F-1-YFP differs from the GT1 reference by 54 unique SNVs. When these numbers are compared to the number of SNVs shared among all three strains (984), this suggests that of all SNVs at least 54/1038 × 100 = 5.2% for 2F-1-YFP2, (85 + 66)/1135 × 100 = 13.3% for B-RH, and (85 + 19)/1088 × 100 = 9.6% for G-RH have accumulated during *in vitro* growth under lab conditions.

We also assessed the impact of the mutations on protein coding (Figure 3B). We observed that 14.3% of the shared variations (141 out of 984) cause a change in amino acid sequence; either a non-synonymous mutation or the generation of a non-sense codon. 47 SNVs cause synonymous mutations. Thus, 19.1% of SNVs caused a mutation within protein coding sequence (141 + 47 out of 984; Figure 3B). The differences between the two RH strains have accumulated during laboratory maintenance, as they originate from the same clonal line [38]. The 85 variations shared between the B-RH and G-RH strains not found in 2F-1-YFP2 suggest that either the parent RH strain used to generate the HXGPRT deletion diverged from the RH strain used to generate the 2F strain, or that the RH-ΔHXGPRT strain accumulated a significant number of mutations before the strain was shared between the Roos and Boothroyd labs.

### ENU mutagenic profile

**Figure 4:**
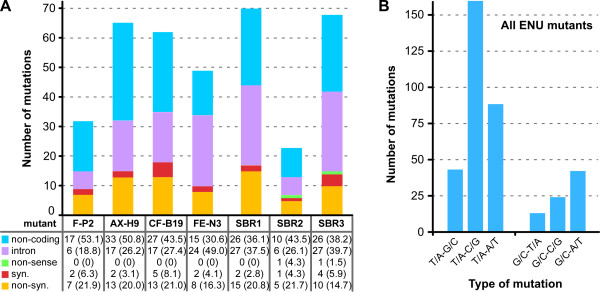
**ENU generated mutants. (A)** The incidence of various SNVs in protein coding regions versus those outside annotated open reading frames. Syn.: synonymous; non-syn: non-synonymous. The number between brackets is the percentage of mutations in each category. **(B)** The incidence of SNVs across all seven ENU mutants in panel **A**. Mutant F-P2 was generated using a 55% killing dose; for all others a dose inducing 70% killing was used. Mutants F-P2, AX-H9, CF-B19, and FE-N3 were generated by Gubbels; mutants SBR1-3 were generated by the Blader lab.

A single ENU mutant was created at a dose resulting in 55% killing (mutant F-P2) and six mutants were derived from a 70% killing dose (all other ENU mutants) (Figure 1). For mutants generated at 70% killing, the number of mutations varies from 23 (SBR2) to 70 (SBR1), with an average of 56.2 (Figure 4A). SBR2 is a likely outlier in the 70% set, lying even below the 55% killing data point (32 SNVs). Omitting SBR2 results in an average of 62.8 SNVs at 70% killing. In either case, the variability at one of only two doses prevents a robust correlation between mutagen dosage and number of mutations.

When we analyze the bases targeted by ENU we find that 78.6% of mutations are found at A or T (Figure 4B). This is quite different from the random nucleotide distribution observed during *in vitro* culture where 44.5% of the SNVs are at A or T (Figure 3D), close to the natural incidence in the genome (45% A or T) [55]. ENU mutagenesis generated a ti/tv ratio of 1.2, slightly higher than observed in the spontaneous mutations (Figure 4B).

### EMS mutagenic profile

**Figure 5:**
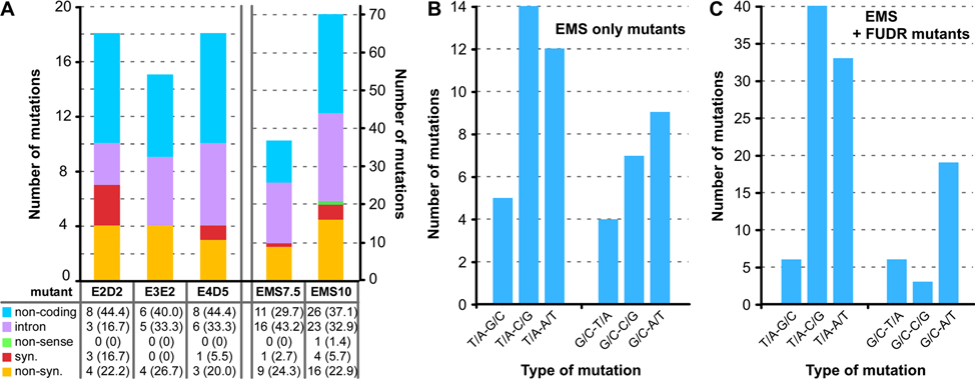
**EMS generated mutants. (A)** The incidence of various SNVs in protein coding regions versus those outside annotated open reading frames. Syn.: synonymous; non-syn: non-synonymous. The number between brackets is the percentage of mutations in each category. **(B)** The incidence of SNVs across the EMS mutants screened for resistance to pharmacologically induced egress using DTT (E2D2 and E4D5) or invasion enhancing compound 2 (E3E2) [42, 43]. These three mutants were generated using an EMS dosage inducing 70% killing. **(C)** The incidence of SNVs across the EMS mutants screened for resistance against 20 μM FUDR [15]. EMS7.5 and EMS10 were generated using 7.5 and 10 mM EMS, inducing 80% and 90% killing, respectively.

We generated EMS mutants at dosages inducing 70%, 80% and 90% killing. In order to inform future genetic studies, we used these data to determine the relationship between the dose of EMS and the number and type of resulting mutations. At 70% killing, the number of SNVs was very consistent, with 18, 15 and 18 mutations identified in E2D2, E3E2 and E4D5, respectively. At 80% killing (mutant EMS7.5) this number increased to 37 SNVs and further increased to 70 SNVs at 90% killing (mutant EMS10) (Figure 5A). These data show that the number of mutations induced by EMS approximately doubles as killing increases by 10%.

Across all EMS mutants 23.4% of the SNVs represented non-synonymous mutations (Figure 5A), which was slightly higher than the 19.8% observed for ENU mutagenesis (Figure 4A). Thus, both chemical mutagens led to increased levels of non-synonymous mutations relative to the 14.3% observed during long term *in vitro* growth (Figure 3B). EMS mutants showed a ti/tv rate of 1.06. This was slightly lower than that of ENU but still higher than the rate observed for spontaneous mutations. Importantly, although the ti/tv rate is lower, the number of non-synonymous mutations is actually higher for EMS compared to ENU.

Following mutagenesis, the 80% and 90% killing samples were selected via induction of resistance to FUDR. This is a nucleotide analogue of deoxyuridine, and could therefore potentially have a mutagenic effect. Uracil phosphoribosyl transferase (UPRT) converts FUDR to fluorodeoxyuridylic acid, a suicide inhibitor of thymidilate synthase [6]. It is therefore possible that usage of FUDR or its metabolic derivatives could result in DNA mutations that skew the mutagenic profile. As such, we considered the base preferences for the EMS mutants separately for those mutants generated in the absence and presence of FUDR selection (Figure 5B and 5C, respectively). The percentage of mutations found at A/T bases was 60.8% for mutants generated with EMS alone (51 total SNVs) and 73.8% for those additionally selected with FUDR (107 SNVs). The ti/tv ratio in the EMS alone mutants was 0.8, while for the FUDR selected strains ti/tv was 1.2. It appears that selection with FUDR may have the potential to influence the EMS mutagenic profile. However, the subtlety of this effect, combined with the small sample size means that any effects of FUDR on the mutagenic profile of our SNV pool are minor. Therefore, the FUDR treated and untreated EMS mutagenized samples have been considered together in our analyses, as the effects of EMS are certain to be the more significant source of mutations than the selection with FUDR (an increase in EMS dosage by 10% in killing rate doubles the number of SNVs regardless of whether FUDR is present).

### Characterization of FUDR resistant parasites

**Figure 6:**
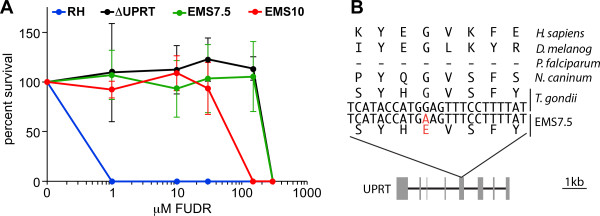
**FUDR resistant mutants. (A)** The resistance of *Toxoplasma* strains to varying concentrations of FUDR. Survival was determined by plaque formation relative to an untreated control. Error bars represent SD from 6 independent experiments. Both strains were initially selected for survival in the presence of 20 μM FUDR. **(B)** Proposed etiological mutation in UPRT identified in mutant EMS7.5. Genbank accession numbers for HsUPRT and DmUPRT are NP_659489 and CG5537, respectively.

Mutants EMS7.5 and EMS10 represent FUDR resistant clones identified by screening against 20 μM FUDR. To more specifically evaluate the FUDR resistant phenotypes we determined the IC50 value of the EMS mutants and compared them with the parent RH strain and parasites wherein the UPRT locus was genetically deleted (ΔUPRT). It has been shown that FUDR resistance can be mediated by mutations in the UPRT gene and that FUDR resistant mutants have no detectable UPRT activity [56]. UPRT functions in the pyrimidine salvage pathway, which is not essential in *Toxoplasma* because it can synthesize its own pyrimidines [57]. The FUDR IC50 for wild type parasites is less than 0.3 μM, and for ΔUPRT the IC50 is 264 μM (Figure 6A), both of which are consistent with data in the literature [10, 58]. Mutant EMS7.5 has an IC50 of 217 μM. Interestingly, mutant EMS10 has an IC50 of 61 μM, which is several fold less than the ΔUPRT strain.

When we analyzed the SNVs in the genomes of these mutants we indeed found a mutation in the UPRT coding sequence in the genome of mutant EMS7.5, which resulted in an amino acid change at position 98 from Gly into Glu (Figure 6B). The conserved nature of this glycine and high level of FUDR resistance in this mutant supports the conclusion that this is a loss of function mutation. Unexpectedly, for mutant EMS10 we did not identify a mutation in the UPRT gene, which may explain the intermediate IC50 of this clone (see Additional file 2: Table S2 for complete list of SNVs in EMS10). While complete mapping of the nature FUDR resistance in this mutant is beyond the scope of this study, these findings highlight the power of chemical mutagenesis and phenotypic screening to identify both expected and unexpected biological targets.

### Genome distribution of mutations

**Figure 7:**
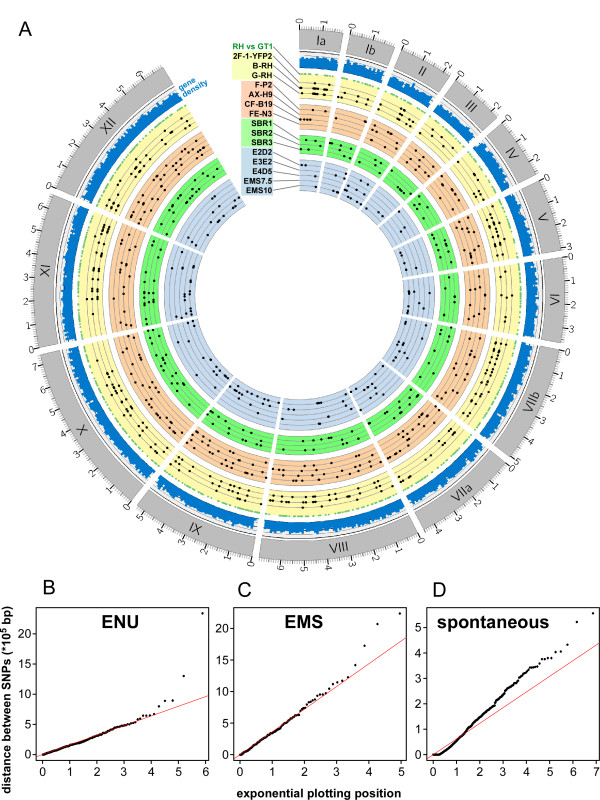
**Genome wide distribution of mutations. (A)** Circos plot of all mutants and gene density. The *Toxoplasma* GT1 reference genome is composed of the 14 chromosomes displayed in the outermost circle. The SNVs shared between all 3 RH parent lines and the GT1 reference are represented by green dots. Strain and mutant specific variations are represented by black dots and are grouped as either parent lines (yellow) or by mutant genealogy and mutagen as shown in Figure 1. Gene density was mapped using a sliding window approach with a window size of 100 kb. **(B-D)** QQ-plot of SNV distances vs. exponential distribution. To test the assumption that mutations are distributed randomly, the inter-SNV distances (taken from Additional file 2: Table S2) are plotted against the exponential distribution, with λ = 1/mean, using a Quantile-Quantile plot; B = distances between ENU induced SNVs (N = 368, mean = 159582), C = distances between EMS induced SNVs (N = 144, mean = 359992.3), D = distances between shared SNVs between the parent strains (N = 968, mean = 61631.86). The solid red line in each plot represents the null assumption *y* = *x.*

The data assembled here comprise 1208 naturally occurring SNVs (Figure 3A) and 527 chemically induced mutations (369 in the ENU mutants, Figure 4; 158 in the EMS mutants, Figure 5). Using circos plots we plotted SNVs on the chromosomes together with the gene density [59] (Figure 7). Despite this small sample size we analyzed these plots for clusters of variants into so-called hot- and cold-spots. We reasoned that hot regions could be low in gene coding sequence and vice versa. Based on these data we do not observe any obvious hot or cold spots for either naturally occurring or chemically induced SNVs, and as such we detect no clear correlation between SNV location and gene density. While we did not observe signatures of bias at any specific regions of the genome, we sought to further test the randomness of the distribution of mutations across the genome as a whole. Randomly distributed mutations follow a Poisson distribution with λ equal to the SNV rate. It thus follows that the distance between randomly distributed SNVs will be exponentially distributed with a rate equal to 1/λ [60]. Quantile-Quantile (QQ) plots of the inter-mutational distances for each sample versus the exponential distribution are plotted in Figure 7B-D. The mutations in the parent samples accumulated during normal *in vitro* culture and have been subject to the selective pressures associated with that environment for numerous generations. As expected these variants do not appear randomly distributed, demonstrated by their divergence from the exponential distribution (Figure 7D). This indicates that these variants are not randomly distributed. Both the ENU- and EMS-derived SNV distributions closely match the theoretical random distribution (Figure 7B, 7C), indicating that the induced random mutations are, indeed, randomly distributed. Using the two-sample Kolmogorov-Smirnov test for each sample against the assumption of an exponential distribution, ENU has p-value = 0.7107 and EMS has p-value = 0.8782 indicating that they do follow the exponential distribution and are thus randomly distributed. The SNVs shared between the parent samples have a p-value = 2.2e-16 indicating that these mutations are not exponentially distributed and thus not random. To assure ourselves that the difference in distribution between the mutagen-derived SNVs and the *in vitro* culture derived SNVs is not due to sample size, we repeatedly downsampled the *in vitro* set to match the number of SNVs in the two mutagenesis data sets and this did not affect the conclusions. Overall, these data show that ENU and EMS induce a random distribution of mutations across all chromosomes, whereas the accumulation of SNVs over time in cell culture is non-random, consistent with selection.

## Discussion

The goal of this study was to better understand the nature of chemical mutagenesis to make informed decisions in the fine-tuning of forward genetic studies in *Toxoplasma*. Additionally we characterized several other salient effects of laboratory manipulation on the *Toxoplasma* genome. We identified significant variation between the supposedly identical lab strains passaged in different labs. Relatively small numbers of completely unique mutations (19–66) were observed when analyzing individual parent strains. However, B-RH and G-RH, which are derived from the same clone, share an additional 85 SNVs not found in the 2F-1-YFP2 strain. These 85 mutations originated before this line was distributed to different labs. These lab-strains were generated and separated 10–15 years ago, which encompasses a variable and unknown number of generations, preventing us from making an accurate estimate of the mutation rate per generation.

We identified almost 1,000 variations among three different versions of the lab adapted RH strain as compared to the reference strain GT1 (Figure 1). This value closely resembles the number of mutations based on a more limited data set [61] and our data further validates this number. The variations observed in the parent strains were non-randomly distributed across the genome, with a bias away from coding regions (Figures 3, 7). This is consistent with the principle of purifying selection, wherein amino acid changes confer a competitive disadvantage [62]. Our data shows a genome-wide ti/tv ratio close to 1 for spontaneous mutations. No previous reports for ti/tv ratios in *Toxoplasma* are available, but this ratio is within the normal range of ratios seen in other organisms [53, 54].

We attempted to determine whether the selection of parasites with different drugs might influence mutagenic profiles (Figure 1). For instance, 6-TX and FUDR are nucleotide analogs that could result in mutations in the genome, whereas phleomycin is a validated mutagen in *Toxoplasma*, causing double-strand breaks [63]. We were unable to detect any definitive mutagenic signatures for any of these drug selections, though a slight decrease in A/T mutation preference was observed in FUDR selected mutants. While the A/T% and ti/tv ratio for 2F-1-YFP2 differed from the other lab strains, it is unlikely that this is due to its selection with phleomycin. Single nucleotide mutations are not an expected consequence of double strand break repair. In our initial analysis we did screen for short indels, however none of the calls reached high confidence values and none could be experimentally validated, likely due to the significant divergence between the RH strain and the GT1 reference. Attempts to include indels called by the current software package increased our false positive rate without contributing any valid calls, and thus indel calling was not used in this analysis. New software packages, which directly compare mutant and parent rather than aligning both to the reference such as NIKS [64] or RUFUS (Farrell, Marth et al., in preparation), will most likely perform better for indels if the reference is of low quality.

Interestingly, we detected 50 polymorphisms between the apicoplast genome of GT1 and the RH strain. Since the apicoplast genome is contained on 35 kb of circular DNA [65] this indicates a 100-fold higher spontaneous mutagenesis rate in the plastid’s genome than in the nuclear genome. This may highlight differences in the fidelity of plastid genome replication or to a unique deployment of DNA repair enzymes in this organelle. Another possible explanation for the high rate of mutations observed in the apicoplast is the polyploidy of the plastid genome. The relative number of sequence reads mapping to the plastid suggest 7, 14, and 20 copies of its genome in B-RH, G-RH, and n-F-P2, respectively. Ploidy of up to 25 N is consistent with previous reports [66, 67], although the variation is quite wide across strains.

Our finding that ~74% of the EMS mutations are at an A/T base pair in *Toxoplasma* (Figure 5) deviates dramatically from the general observation across other systems where EMS has a GC-biased signature [21–23]. The mechanism of EMS mutagenic activity is the reaction of its ethyl group predominantly with guanine and to a much lower extent with adenine to generate N-alkyl adducts [68]. During DNA replication, DNA polymerases frequently place thymine, instead of cytosine, opposite ethylguanine resulting in transitions. The presence of 3-alkyl-adenine is not immediately mutagenic in mammalian cells [69]. It has been suggested that this could be due to an SOS-like response turned on by cytotoxic lesions like 3-alkyladenine, or, alternatively, that increased removal of 3-alkyladenine increases the number of single-strand breaks in DNA, which stalls DNA replication and allows a prolonged time for DNA repair by the alkyltransferase [69]. It is currently neither known how *Toxoplasma* DNA polymerase handles different kinds of DNA damage, nor whether *Toxoplasma* mounts an SOS response in response to DNA damage. *Toxoplasma* possesses two apurinic/apyrimidinic (AP) endonucleases that function in DNA base excision repair: exonuclease III (TgAPE) and endonuclease IV (TgAPN) [70] but their activities against ENU or EMS induced damage have not been determined. Collectively, these findings indicate that the distinct EMS mutagenic signature in *Toxoplasma* is most likely caused by a difference in DNA repair pathways and suggest that *Toxoplasma* is able to repair G-alkylation quite well and A-alkylation very poorly. The signature of mutagenesis by both EMS and ENU clearly differs from the accumulation of SNVs through normal *in vitro* culture, where the A/T bias nearly mirrors the 45% A/T composition of the genome as a whole.

One of the new insights generated here is the relationship between particular dosages of ENU or EMS (as measured by percent killing) and the total number of resultant mutations. In the past, estimates of this number have been made experimentally using pro-drugs like 6-TX and FUDR targeting the negative selectable markers HXGPRT and UPRT, respectively [8, 11, 15]. This led to an estimated 10–100 mutations per genome (65 Mb; haploid) using an ENU dosage inducing 70% killing, but since it was unknown which mutations resulted in drug resistance these estimates were very rough. The data shown here confirm that these estimates were accurate, as we always observe less than 100 SNVs per genome at the highest dosage used (70% ENU killing and 90% EMS killing). This approximates 1 mutation per Mb, which appears to be the “magic number” as it is consistently observed in mutagenesis studies that aim to generate phenotypic mutants across organisms. This includes ENU mutagenized mice (roughly 1 mutation per Mb [71]), a combination of mutagens in the yeast *Pichia stipites* (14 mutations in the 15 Mb genome [25]), and EMS mutagenesis of the yeast *Saccharomyces cerevisiae* (1 mutation per Mb [72]).

We also generated FUDR resistant mutants in this study as an internal control for mutagenesis experiments at high EMS dosages. Mutants EMS7.5 and EMS10 were treated with 20 μM FUDR to select for mutations in the UPRT gene (Figures 5, 6). The Gly98 into Glu UPRT mutation in EMS7.5 is consistent with resistance to FUDR since this residue lies in β-arm of the dimerization interface, and the crystal structure indicates that dimerization is required for substrate binding and enzymatic activity [73]. However, in mutant EMS10 we did not identify a mutation in UPRT. This mutant is also less resistant to FUDR than the complete UPRT gene deletion or mutant EMS7.5 (Figure 6), hinting at a potentially different resistance mechanism. The list of non-synonymous variations contains many hypothetical genes, which could potentially be involved in FUDR resistance, but the list also contains CPSII, which is the first enzyme in the de novo pyrimidine synthesis pathway (Lys1327 into Arg). It is known that the cytotoxicity of FUDR and 5-FU can be pluriform and the details are poorly understood [74]. Whether the mutation in CPSII indeed confers FUDR resistance is outside the scope of the current study and was not further pursued here.

Our data show that chemical mutagenesis with either ENU or EMS induces mutations randomly distributed over the genome (Figure 7). From a practical forward genetic perspective that is the desired situation. Because of its use in Pfefferkorn’s early experiments, ENU became the mutagen of choice in *Toxoplasma*[5]. In this study we confirm Pfefferkorn’s later findings that efficient mutagenesis was also obtained with EMS treatment of actively growing intracellular parasites ( [15]; nitrosoguanidine treatment of extracellular parasites was on par with EMS). We also show that, compared to ENU, EMS produces a similar or slightly higher fraction of non-synonymous mutations, which is preferred in chemical mutagenesis experiments for mutant screens. From a practical perspective, the ideal number of SNVs for genetic studies in *Toxoplasma* is 5–10 non-synonymous mutations per genome [7, 13, 33]. To be within this range, the optimal dosage of EMS based on our data would be an EMS concentration inducing 80% killing (Figure 5). However, we would like to note this recommendation is based on only two data points at 80% and 90% killing causing 9 and 17 non-synonymous mutations, respectively. If for other experimental considerations ENU should be the preferred mutagen, we recommend an ENU dosage inducing 60% killing to be within the 5–10 non-synonymous mutation range.

## Conclusions

We successfully applied whole genome sequencing and mutational profiling methods to *Toxoplasma* and identified genome variations acquired during long-term *in vitro* culture and upon chemical mutagenesis. Comparison of the three different lab strains showed that serial passaging of parasites over 10–15 years has resulted in the accumulation of 50–100 SNVs, predominantly in non-coding sequence or as silent mutations with respect to amino acid code. This is the first time we are able to provide insights on the accumulation of polymorphisms in cultured strains of *T. gondii*, which are important variables that could underlie potential differences in experimental outcome in different labs. Although the experiments here were not explicitly designed to detect mutagenic profiles for different drug selectable markers, a subtle change in A/T bias was detected following FUDR selection in EMS-generated mutants. No such signatures were identified for 6-TX or phleomycin selection, suggesting any mutagenic effects of drug selection are minimal in comparison to those generated by the underlying mutagen.

As in other organisms, the ENU mutagenic profile in *Toxoplasma* showed a consistent A/T bias. Interestingly, a similar A/T bias was observed with EMS, which represents a stark departure from the neutral or G/C bias commonly observed in other systems. This suggests the DNA repair of EMS damage in *Toxoplasma* differs from other well-studied genetic systems. Of importance for the design and interpretation of forward genetic experiments, we validated that both ENU and EMS induce mutations randomly over the genome. For EMS we observed a slightly higher rate of non-synonymous point mutations and a lower rate of mutation in non-coding sequence compared to ENU. These differences, while subtle, make EMS more likely to generate coding changes while minimizing non-coding SNVs that can be challenging to functionally dissect. EMS is therefore a preferable mutagen for forward genetic experiments where non-synonymous mutations are the desired outcome. From our experience, an 80% killing dose of EMS, which yields approximately 35 SNVs, will cause 5–10 coding changes. Following phenotypic selection, this is a feasible number of genes upon which to focus. We believe that the insights presented here will not only be helpful to studies in the relatively well-accessible *Toxoplasma* parasite, but will pave the way toward forward genetic studies in less accessible protozoan pathogens as well.

### Availability of supporting data

Sequence reads have been deposited at the National Center for Biotechnology Information sequence read archive under accession numbers SRR346134 (2F-1-YFP2), SRR346136 (o-F-P2), SUB465503 (n-F-P2), SUB46416 (B-RH), SUB464162 (G-RH) and SAMN02647169 - SAMN02647179 for all other strains.

## Electronic supplementary material

Additional file 1: Table S1: SNV validation materials and results for mutants E2D2, E3E2, and E4D5. Given are the chromosomal localization of the SNV call, primer sequences used for PCR amplification of predicted SNV from mutant and parent line, and whether the SNV validated by Sanger sequencing of the PCR products. (XLSX 50 KB)

Additional file 2: Table S2: SNV calling and annotation data for all mutants and parent lines. Includes all data presented in Figures 3, 4, 5, and 7. Depths reported are the total read coverage at the given location for Parent and Mutant sample in each experiment respectively. Prob is the phred scaled probability of the variation as reported by FREEBAYES. (XLSX 358 KB)

## Abbreviations

6-TX: 6-ThioXanthine
bp: base pair
Chl: Chloramphenicol
ENU: N-ethyl-N-nitrosourea
EMS: Ethylmethane sulfonate
FUDR: 5-fluoro-2'-deoxyuridine
HFF: Human foreskin fibroblast
HXGPRT: Hypoxanthine-xanthine-guanine phosphoribosyltransferase
Phle: Phleomycin
SNV: Single nucleotide variation
UPRT: Uracil phosphoribosyl transferase.
